# US-Elastography With Different Techniques for Thyroid Nodule Characterization: Systematic Review and Meta-analysis

**DOI:** 10.3389/fonc.2022.845549

**Published:** 2022-03-16

**Authors:** Vito Cantisani, Annalisa De Silvestri, Valeria Scotti, Daniele Fresilli, Maria Grazia Tarsitano, Giorgia Polti, Olga Guiban, Eleonora Polito, Patrizia Pacini, Cosimo Durante, Giorgio Grani, Andrea M. Isidori, Elisa Giannetta, Salvatore Sorrenti, Pierpaolo Trimboli, Carlo Catalano, Roberto Cirocchi, Augusto Lauro, Vito D’Andrea

**Affiliations:** ^1^ Department of Radiological Sciences, Oncology and Pathology, Policlinico Umberto I, Sapienza University of Rome, Rome, Italy; ^2^ Servizio di Epidemiologia Clinica e Biometria Direzione Scientifica—Fondazione Istituti di Ricovero e Cura a Carattere Scientifico (IRCCS) Policlinico san Matteo, Pavia, Italy; ^3^ Department of Clinical and Surgical Science, “Magna Graecia” University, Catanzaro, Italy; ^4^ Department of Translational and Precision Medicine, “Sapienza” University of Rome, Rome, Italy; ^5^ Department of Experimental Medicine, Sapienza University of Rome, Rome, Italy; ^6^ Department of Surgical Sciences, Hospital “Policlinico Umberto I”, “Sapienza” University of Rome, Rome, Italy; ^7^ Clinic for Endocrinology and Diabetology, Lugano Regional Hospital, Ente Ospedaliero Cantonale, Lugano, Switzerland; ^8^ Department of Surgery and Biomedical Sciences, University of Perugia, Perugia, Italy

**Keywords:** thyroid, USE, shear wave elastography, strain elastography, meta-analysis, real-time elastography

## Abstract

**Background:**

Thyroid nodules are frequent in adult population and thyroid cancer incidence has increased dramatically over the past three decades. The aim of this systematic review and meta-analysis was to evaluate the US-Elastosonography (USE) diagnostic performance in assessing the thyroid nodules malignancy risk.

**Methods:**

PubMed and Embase databases were searched from January 2011 to July 2021. We extracted data from selected studies and calculated the overall diagnostic accuracy of qualitative USE, semi-quantitative USE and quantitative USE. Summary receiver operating characteristic (ROC) curve was elaborated to show the results. All statistical tests were performed using Metadisc and Medcal software package.

**Results:**

Finally 72 studies with 13,505 patients and 14,015 thyroid nodules (33% malignant) undergoing elastography were included. The pooled sensitivity, specificity and AUC were 84%, 81%, and 0.89 respectively for qualitative USE; 83%, 80%, and 0.93 for semi-quantitative USE and 78%, 81% and 0.87, for quantitative USE. The qualitative and semiquantitative USE present very similar diagnostic accuracy values and both better than the quantitative USE.

**Conclusions:**

USE is a useful imaging tool for thyroid nodule characterization. In accordance with recent guidelines and meta-analyses, the USE could be used daily in thyroid nodule malignancy risk stratification.

**Systematic Review Registration:**

PROSPERO: CRD42021279257.

## Introduction

Thyroid nodules are frequent in adult population up to 60%, with a prevalence of cancer as 5% (1, 2). Since the incidence of thyroid cancer has mostly increased in the last decade (3, 4) the initial assessment of these patients is a hot topic and ultrasound (US) represents the first line imaging modality in this context. In fact, the US features such as micro- or macrocalcifications, marked hypo echogenicity, taller than wide shape, and thick irregular or lobulated margins are recognized as associated with malignancy (5), but they are not highly predictive: US sensitivity and specificity have high variability ranging between 52 and 97% and 26.6 and 83%, respectively. In addition, low reproducibility and operator-depending performance might reduce US diagnostic value. Thus, the only US images are suboptimal to actually diagnose a thyroid cancer.

To reduce or delete these limitations, several Thyroid Imaging Reporting and Data Systems (TIRADS) (6–9) have recently been proposed as a tool for uniform reporting and consistent evaluation.

This risk stratification should guide the indication for fine needle aspiration biopsy (FNAB) that is required when a suspicious nodule is identified, with normal thyroid stimulating hormone. FNAB presents a specificity of 60 to 98% and sensitivities from 54 to 90% (10, 11), so it is not such an accurate exam. In fact, non-diagnostic and indeterminate responses are common (12–15). Consequentially, on one hand, a significant number of patients have to repeat the procedure with incremented costs and on the other hand, some patients could receive unnecessary thyroid surgery, more for diagnostic than for therapeutic purposes. Considering these points and the known risks of thyroid surgery, improving the techniques for thyroid nodules diagnosis is mandatory. Among the different techniques, in the present paper we will address the role of Ultrasound elastography (USE) for thyroid characterization. Based on the fact that a suspicious nodule is at palpatory firm or hard in consistency, stiffness was adopted as indicator of malignancy for elastography (16, 17).

In this way, USE was utilized, and by the beginning encouraged literature data were obtained and as a consequence it was suggested very soon as an additional tool for thyroid nodule differentiation, in combination with conventional US and FNAB (18, 19).

Consequently, USE methods have been incorporated into international guidelines published by the WFUMB (World Federation for Ultrasound in Medicine and Biology) (20) and the EFSUMB (European Federation of Societies for Ultrasound in Medicine and Biology) (21); in the above-mentioned Guidelines, technical details, advantages and limitations for strain elastography (SE) and quantitative 2 D ultrasound shear wave elastography (SWE) have extensively been reported.

However, technology improvements, and open issues already reported by guidelines were reported to be addressed. To the best of our knowledge, presently, few studies have investigated the diagnostic performance of various thyroid Ultrasound elastography (UE) methods as applied in the clinical context and shown variable results.

Hence, this present, updated systematic review (registered in the international prospective register of systematic reviews PROSPERO: CRD42021279257) and meta-analysis assesses and summarizes current evidence on the diagnostic performance of various thyroid USE software in differentiating benign and malignant thyroid nodules.

## Materials and Methods

### Literature Search

The following electronic databases were searched: PubMed and EMBASE.

The search strategy was based on the PICOS framework to identify search key words relating to the population, intervention, and outcomes in the different databases. The search concepts were: 1. Thyroid nodule AND 2. ultrasound 3. elastography OR elastosonography 4. SWE OR Shear wave elastography, 5. Strain 6. ARFI OR acoustic radiation force, and their related terms as MeSH terms, keywords and/or EmTree terms.

The search was conducted between January 2011 and July 2021 and only in English language.

### Inclusion and Exclusion Criteria

From all retrieved references, duplicates were eliminated and the remaining records were screened.

All references identified were independently assessed by two authors, first by means of title and abstract, then by the review of the complete paper.

All the studies analyzed had to meet the following criteria: 1. The study involved only human subjects; 2. The study investigated the diagnostic performance of USE techniques as Strain and Shear wave for differentiation of benign and malignant thyroid nodules in a clinical setting; 3. Use of an appropriate reference standard (FNAC or histopathology); and 4. Diagnostic performance outcomes of interest were reported in terms of sensitivity, specificity, negative predictive values (NPV), positive predictive values (PPV), diagnostic accuracy, and/or area under receiver operator characteristic curve ROC curve (AUROC).

Exclusion criteria were: 1. Case reports, editorial letters, or commentaries; 2. Studies that included less of 50 thyroid nodules 3. Non-English; and 4. Insufficient diagnostic accuracy outcomes and studies without values of sensitivity, specificity, NPV and PPV; 5. paper related to specific categories such as Indeterminate nodules at Cytology.

### Data Extraction

Two independent readers extracted the data in a pre-specified form. For each article, the following data were extracted: bibliographic data, type of study, type of setting, number of patients, demographic/clinical data (age, type of lesions, percentage of men and women), and number of nodules and prevalence of malignant nodules. Furthermore, for each USE techniques true (TP) and false positive (FP), true (TN) and false negative (FN) were retrieved or calculated from sensitivity/specificity.

### Quality Assessment

The quality of the studies included in the meta-analysis was assessed with a checklist based on the Quality Assessment for Studies of Diagnostic Accuracy (QUADAS 2) tool (22). Two investigators performed a quality assessment of the included studies independently, and disagreements were resolved by discussion.

### Data Analysis Approach

The statistical pooling of test accuracy studies presents an added level of complexity as accuracy is usually quantified by two related statistics (sensitivity and specificity) rather than one, and meta-analysis must allow for the trade-off between the two. Positive and negative likelihood ratios (LRs) (that allow for this trade-off) were pooled with weighted averages applied, in which the weight of each study was its sample size. For each pooled estimate, a 95% confidence interval (CI) was calculated using random effects model. Positive and negative LRs (representing likelihood of malignancy in case of positive or negative results of index USE technique) could be interpreted as in **Table 1** (23).

**Table 1:** Interpretation of the LR.

LR	Effect on Post-test Probability of Disease	Comment
Values between 0 and 1 decrease the probability of disease		
0.1	Large decrease	Conclusive
0.2	Moderate decrease	Useful
0.5	Slight decrease	Moderately useful
1	None	Not useful
Values >1 increase the probability of disease		
1	None	Not useful
2	Slight increase	Moderately useful
5	Moderate increase	Useful
10	Large increase	Conclusive

A symmetric summary receiver operating characteristic (ROC) curve, as described by Moses et al. (24) was constructed to summarize the results; the area under this curve (AUC) was calculated.

Study heterogeneity was assessed by the I^2^ index, which describes the percentage of total variation across studies that is due to heterogeneity rather than chance. A value of greater than 50% may be considered indicative of significant heterogeneity.

AUCs were compared with a z-test of the ratio between difference of AUC and square root of the variance of the difference (25).

Furthermore, a sub-analysis regarding prospective and retrospective papers was carried out.

All statistical tests were performed using Metadisc (26) and Medcal (27) software package.

## Results

We retrieved 437 records (113 in PubMed and 324 in Embase) that were 353 after removing the duplicates; of them 72 full-text were carefully examined and all of them, from whom TP, FP FN and TN were retrievable for single USE techniques, were included in meta-analysis. Quality of studies was generally high. Mean age of the 13,505 patients was 46 years; mean percentage of men was 24%.

The total thyroid nodules included in our study was 14,015.

A high malignancy rate (33%) was observed compared to the general population and with a pooled malignancy of 32% for qualitative USE, 29% for semi-quantitative USE and 33% for quantitative USE. The pooled sensitivity, specificity and AUC were 84% (95% confidence interval (CI), 0.83–0.85), 81% (95% CI, 0.80–0.82) and 0.89 (95% CI, 0.87–0.91) respectively for qualitative USE; 83% (95% CI, 0.81–0.84), 80% (95% CI, 0.79–0.82) and 0.93 (95% CI, 0.91–0.95) respectively for semi-quantitative USE and 78% (95% CI, 0.76–0.79), 81% (95% CI, 0.80–0.82) and 0.87 (95% CI, 0.86–0.88), respectively for quantitative USE. The positive likelihood ratios (PLR) and negative likelihood ratios (NLR) were 4.7 (95% CI, 3.5–6.3) and 0.24 (95% CI, 0.17–0.34) for qualitative USE; 6.5 (95% CI, 4.2–10.1) and 0.19 (95% CI, 0.13–0.27) for semi-quantitative USE; 4.4 (95% CI, 3.6–5.5) and 0.28 (95% CI, 0.24–0.33) for quantitative USE.

**Figure 1:**
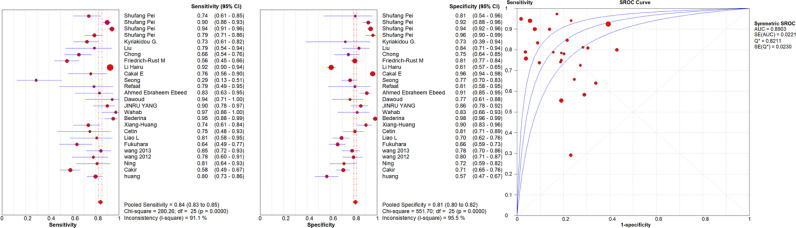
Sensitivity and specificity forest plots and SROC curve for the qualitative USE. The circle size represents the nodule population of the selected articles; the line represents the confidence interval of the selected articles.

**Figure 2:**
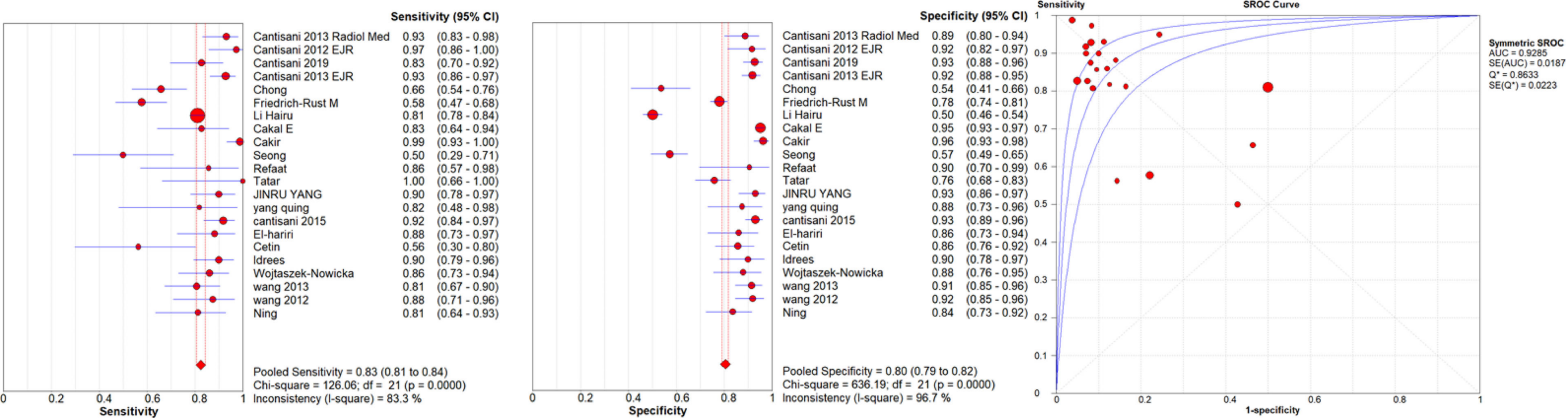
Sensitivity and specificity forest plots and SROC curve for the semiquantitative USE. The circle size represents the nodule population of the selected articles; the line represents the confidence interval of the selected articles.

**Figure 3:**
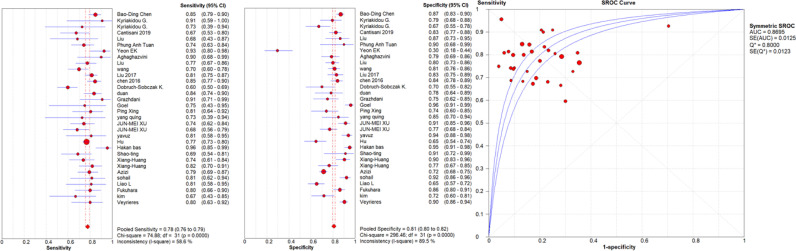
Sensitivity and specificity forest plots and SROC curve for the quantitative USE. The circle size represents the nodule population of the selected articles; the line represents the confidence interval of the selected articles.

The results are synthesized in **Tables 2**–**4** and **Figures 1**–**3**.

**Table 2:** PRISMA flow diagram of articles included.

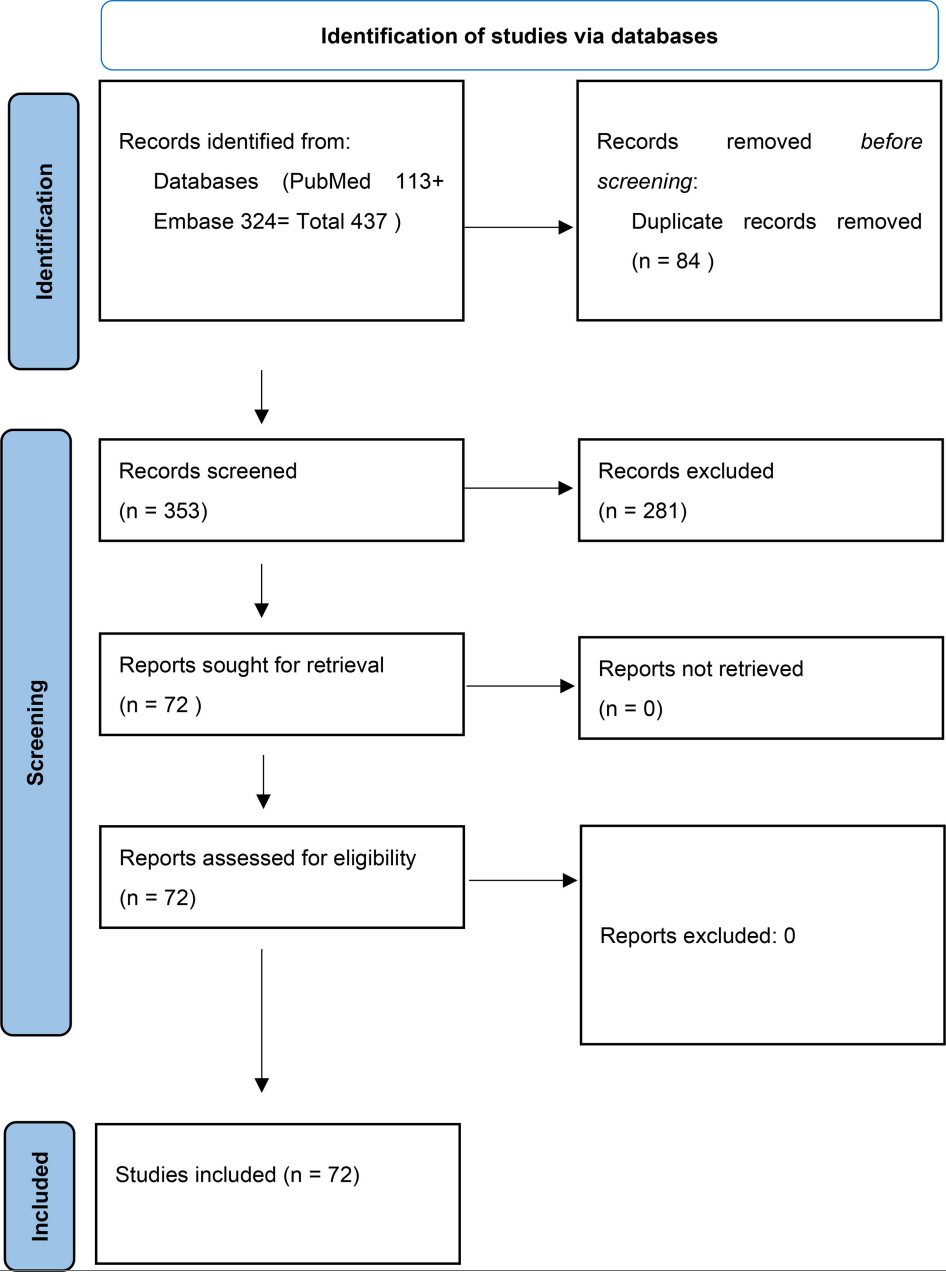

**Table 3:** Included studies details.

First author	Year	Elastography	Estimate of sensitivity	Estimates of specificity	Prospective	Retrospective
Chong Y. (28)	2013	qualitative USE	66%	75%	x	
		semi-quantitative USE	66%	54%		
Cantisani V. (29)	2013	semi-quantitative USE	93%	92%	x	
Refaat R. (30)	2014	qualitative USE	79%	81%	x	
		semi-quantitative USE	86%	90%		
Tatar I.G. (31)	2014	semi-quantitative USE	100%	76%	x	
Grazhdani H. (32)	2014	quantitative USE	91%	75%	x	
Mona A. (33)	2014	semi-quantitative USE	88%	86%	x	
Jun-Mei X. (34)	2014	qualitative USE	74%	91%		x
		quantitative USE	68%	77%		
Cakir B. (35)	2014	semi-quantitative USE	99%	96%	x	
Bederina E.L. (36)	2014	qualitative USE	95%	98%	x	
Erman Çakal. (37)	2014	qualitative USE	76%	96%	x	
		semi-quantitative USE	83	95		
Cantisani V. (38)	2015	semi-quantitative USE	92%	93%	x	
Huang X. (39)	2015	qualitative USE	74%	90%		x
		quantitative USE	82%	77%		
		quantitative USE	74%	90%		
Azizi G. (40)	2015	quantitative USE	79%	72%	x	
Cetin N. (41)	2015	semi-quantitative USE	56%	86%	x	
		qualitative USE	75%	81%		
Dobruch-Sobczak K. (42)	2016	quantitative USE	60%	70%	x	
Duan S.B. (43)	2016	quantitative USE	84%	78%	x	
Friedrich-Rust M. (44)	2016	qualitative USE	56%	81%	x	
		semi-quantitative USE	58%	78%		
Ping X. (45)	2016	quantitative USE	81%,	74%	x	
Seong M. (46)	2016	qualitative USE	29%	77%		x
		semi-quantitative USE	50%	57%		
Chen B.D. (47)	2016	quantitative USE	85%	87%		x
Ahmed E.E. (48)	2017	qualitative USE	83%	91%,	x	
Mohammed M.D. (49)	2017	qualitative USE	94%	77%	x	
Liu B.J. (50)	2017	quantitative USE	77%	80%	x	
Liu Z. (51)	2017	quantitative USE	81%	83%		x
Wang D. (52)	2017	quantitative USE	70%	81%		x
Kyriakidou G. (53)	2018	qualitative USE	73%	73%	x	
		quantitative USE	91%	79%		
		quantitative USE	73%	67%		
Wahab S. (54)	2018	qualitative USE	97%	83%	x	
Cantisani V. (55)	2019	semi-quantitative USE	83%	93%	x	
		quantitative USE	67%	83%		
Huang Y. (56)	2019	qualitative USE	80%	57%	x	
** **Yang Q. (57)	2019	quantitative USE	73%	85%	x	
		semi-quantitative USE	82%	88%		
Aghaghazvini L. (58)	2020	quantitative USE	90%	79%	x	
Li H. (59)	2020	qualitative USE	92%	61%		x
		semi-quantitative USE	81%	50%		
Huang S.T. (60)	2020	quantitative USE	69%	91%		x
Goel S. (61)	2020	quantitative USE	75%,	96%	x	
Shufang P. (62)	2020	qualitative USE	74%	81%		x
		qualitative USE	90%	92%		
		qualitative USE	94%	94%		
		qualitative USE	79%	96%		
Yavuz A. (63)	2020	quantitative USE	81%	94%	x	
Jinru Y. (64)	2017	qualitative USE	90%	86%		x
		semi-quantitative USE	90%	93%		
Yeon E.K. (65)	2020	quantitative USE	93%	30%		x
Tuan P.A. (66)	2020	quantitative USE	74%	90%	x	
Hu L. (67)	2021	quantitative USE	77%	65%	x	
Cantisani V. (68)	2012	semi-quantitative USE	97%	92%	x	
Cantisani V. (69)	2013	semi-quantitative USE	93%	89%	x	
Sohail S. (70)	2020	quantitative USE	81%	92%	x	
Idrees A. (71)	2020	semi-quantitative USE	90%	90%		x
Liao L.J. (72)	2019	qualitative USE	81%	70%	x	
		quantitative USE	81%	65%		
Fukuhara T. (73)	2018	qualitative USE	64%	66%	x	
		quantitative USE	80%	86%		
Wojtaszek-Nowicka M. (74)	2017	semi-quantitative USE	86%	88%	x	
Kim H. (75)	2013	quantitative USE	67%	72%		x
Wang H. (76)	2013	qualitative USE	85%	78%	x	
		semi-quantitative USE	81%	91%		
Wang H.L. (77)	2012	qualitative USE	78%	80%	x	
		semi-quantitative USE	88%	92%		
Veyrieres J.B. (78)	2012	quantitative USE	80%	90%	x	
Ning C.P. (79)	2012	qualitative USE	81%	72%	x	
		semi-quantitative USE	81%	84%		
Cakir B. (80)	2011	qualitative USE	58%	71%	x	
Hakan B. (81)	2021	quantitative USE	96%	95%	x	
Chen M. (82)	2016	quantitative USE	85%	84%		x
Liu B.X. (83)	2014	qualitative USE	79%	84%		x
		quantitative USE	68%	87%		

USE, Ultrasound Elastography.

**Table 4:** Diagnostic performance of each USE methods.

Method	Studies,n	Benign,n	Malignant,n	Sens, %(CI, %)(I-square, %)	Spec, %(CI, %)(I-square, %)	PLR (CI)	NLR (CI)	AUC (CI, %)
Qualitative USE	26	4,635	2,640	84 (83–85) (91.1)	81 (80–82) (95.5)	4.7 (3.5–6.3)	0.24 (0.17–0.34)	89.0 (86.8–91.2)
Semi-quantitative USE	22	3,801	1,889	83 (81–84) (83.3)	80 (79–82) (96.7)	6.5 (4.2–10.1)	0.19 (0.13–0.27)	92.9 (91.0–94.7)
Quantitative USE	32	4,236	2,507	78 (76–79) (58.6)	81 (80–82) (89.5)	4.4 (3.6–5.5)	0.28 (0.24–0.33)	87.0 (85.7–88.2)

USE, Ultrasound Elastography.

Furthermore, a sub-analysis regarding prospective and retrospective works was carried out **(** **Tables 5**, **6**) showing followings results:

o from the prospective studies analysis resulted a pooled sensitivity, specificity and AUC of 74.4%, 82.3%, and 0.87 respectively for qualitative USE; 83.9%, 87.8%, and 0.94 respectively for semi-quantitative USE and finally, 78.0%, 80.9%, and 0.88 respectively for quantitative USE;
o from the retrospective studies analysis resulted a pooled sensitivity, specificity and AUC of 89.0%, 79.7%, and 0.92 respectively for qualitative USE and 78.7%, 81.8%, and 0.87 respectively for quantitative USE.

**Table 5:** Pooled diagnostic performance of qualitative USE using only prospective papers. USE, Ultrasound Elastography.

Method	Sens, %(CI, %)(I-square, %)	Spec, %(CI, %)(I-square, %)	PLR(CI)(I-square, %)	NLR(CI)(I-square, %)	AUC(CI, %)
Qualitative USE	74.4 (71.5–77.2) (81.7)	82.3 (80.9–83.7) (94.7)	4.10 (2.98–5.66) (90.7)	0.30 (0.22–0.40) (79.8)	87.1 (84.1–90.1)
Semi-quantitative USE	83.9 (81.2–86.2) (83.9)	87.8 (86.5–88.9) (90.6)	7.36 (4.78–11.34) (92.6)	0.16 (0.10–0.27) (88.4)	94.3 (92.8–0.95.8)
Quantitative USE	78.0 (76.1–79.8) (55.1)	80.9 (79.5–82.2) (88.6)	4.60 (3.71–5.70) (84.0)	0.26 (0.22–0.32) (63.7)	87.8 (86.3–89.2)

**Table 6:** Pooled diagnostic performance of qualitative USE using only retrospective papers.

Method	Sens, %(CI, %)(I-square, %)	Spec, %(CI, %)(I-square, %)	PLR(CI)(I-square, %)	NLR(CI)(I-square, %)	AUC(CI, %)
Qualitative USE	89.0 (87.5–90.4) (92.1)	79.7 (77.7–81.6) (96.8)	6.10 (2.81–13.24) (96.2)	0.20 (0.10–0.39) (96.4)	92.3 (89.3–95.3)
Quantitative USE	78.7 (76.0–81.2) (63.1)	81.8 (79.8–83.8) (89.3)	4.26 (2.71–6.68) (93.8)	0.28 (0.23–0.34) (58.1)	86.5 (84.7–88.2)

Retrospective papers analysis about semiquantitative USE was not performed because too few papers. USE, Ultrasound Elastography.

The retrospective papers analysis on semi-quantitative USE was not carried out due to the few papers.

The area under the SROC curve was higher than 90% only for semi-quantitative USE (p = 0.19 for semi-quantitative USE vs qualitative USE; p = 0.41 for quantitative USE vs qualitative USE; p = 0.01 quantitative USE vs semi-quantitative USE).

The USE techniques with higher PLR (according to **Table 1** classification could be judged as useful) and lower NLR (according to **Table 1** classification could be judged as useful) was the semi-quantitative USE. Regarding the single dimensions of accuracy, the pooled specificity is equal among USE techniques while sensitivity is lower in quantitative USE than in strain elastography.

The qualitative and semiquantitative USE present very similar diagnostic accuracy values but both better than the quantitative USE. In particular, semi-quantitative USE AUC was statistically higher than quantitative USE one (p-value <0.05).

## Discussion

In addition to the clinical-laboratory evaluations, the clinical–therapeutic management of thyroid nodule is based on the ultrasound examination, which is the preferred thyroid imaging modality due to its non-invasiveness, wide availability and low cost. Several ultrasound features are used to classify thyroid nodules, each of them carrying a more or less high risk of malignancy (84). Trying to standardize the ultrasound estimate of thyroid nodules malignancy risk, it was introduce a risk-score called TIRADS (8, 19, 85, 86). The TIRADS lexicon is based on echo structure (solid, mixed or cystic), echogenicity (hyper, iso, hypoechoic or markedly hypoechoic), margins (regular, microlobulated; irregular/spiculate), internal components (micro or macro calcifications; cystic areas), and the shape [oval; taller than wide (87)] on ultrasound evaluation.

The main advantage of the routine TIRADS use is to identify with a great accuracy suspected thyroid nodules worthy of cytological investigation (88) and to exclude those not deserving at that time, thus reducing the total number and costs of FNA procedures (88, 89). However, TIRADS have limitations: there are many and different TIRADS with similar but non-overlapping classifications, accuracy is far less than 100% they are rarely used in real-life practice [in about 27.2% of the Italian reports (90)]. Therefore, fine-needle aspiration cytology (FNAC) still represents the gold-standard technique for classification of thyroid nodules, due to its high specificity (60–98%) to identify malignant thyroid nodules, but with variable sensitivity (54–90%) (10–14, 91).

In the last decades, many studies and meta-analyzes have demonstrated the effectiveness of new ultrasound techniques such as CEUS (contrast ultrasound) and, above all, USE (US-Elastography) to improve the B-mode assessment of the thyroid nodule (38, 55, 92–100).

Recently USE was introduced in the last guidelines as an additional tool for stratifying the thyroid nodules malignancy risk, in combination with conventional US and FNA. In particular, the EFSUMB (European Federation of Societies for Ultrasound in Medicine and Biology) guidelines assert that Strain Ratio Elastography (SRE) should be part of the thyroid work-up due to its high diagnostic accuracy (92, 93).

The WFUMB guidelines (World Federation for Ultrasound in Medicine and Biology) state that both qualitative and semi-quantitative USE can be used for the evaluation of thyroid nodules and in particular qualitative USE which improves the B-mode ultrasound specificity but semi-quantitative USE is more easily learned (20). Furthermore, they state that SWE also improves the conventional US specificity, particularly in subcentimeter thyroid nodules (20).

Already several papers and meta-analyses assert that US-elastography is superior or similar to conventional ultrasound, in particular the following studies:

– in 2015, Nell et al. published a meta-analysis with 20 articles and 3,908 thyroid nodules assessed by qualitative USE using Asteria elastography (ES) classification. They showed a sensitivity and specificity of 85 and 80% respectively, using an elasticity score threshold between 2 and 3, and a sensitivity and specificity of 99 and 14% respectively, using an elasticity score threshold between 1 and 2. In conclusion they affirm that qualitative elastography can detect benign nodules with a high accuracy (101);
– in 2014, Ghajarzadeh et al. published a metanalysis with 12 articles and 1,180 thyroid nodules assessed by qualitative US-elastography. They showed a sensitivity and specificity of 86 and 66.7% respectively, using an elasticity score threshold between 2 and 3, and a sensitivity and specificity of 98.3 and 19.6% respectively, using a elasticity score threshold between 1 and 2. In conclusion they affirm that USE could be used as thyroid nodule screening tool (102).

Almost in parallel, articles began to be published comparing qualitative and semi-quantitative USE and in particular:

– in 2016, the metanalysis of Tian showed the better SRE accuracy than qualitative USE, with a sensitivity and specificity of 86.5% vs. 81.8% and 86.6% vs. 81.7% respectively (103);
– in 2014, Sun et al. published a metanalysis with 31 papers and 6,544 thyroid nodules assessed by real-time ultrasound elastography. They showed the better SRE accuracy than qualitative USE with a sensitivity and specificity of 85% vs 79% and 80% vs 77% respectively. In conclusion they affirm that the SRE and qualitative USE accuracy are similar although SRE diagnostic value is slightly higher than elasticity score (104);
– in 2013, Razavi et al. published a metanalysis with 24 papers and 3,531 thyroid nodules (2,604 benign and 927 malignant) assessed by qualitative and semiquantitative USE. They showed the better accuracy of SRE assessment than elasticity score evaluation with a sensitivity of 89% vs. 82%, respectively, but with same specificity (82%) (105).

After the introduction of new elastosonographic techniques based on shear wave speeds, new studies and various meta-analyses were published to evaluate the SWE diagnostic performance compared to gold-standards, in particular:

- in 2015, Zhan et al. published a meta-analysis with 16 papers and 2,436 thyroid nodules (1,691 benign and 745 malignant) assessed by ARFI (acoustic radiation force impulse) imaging. They showed a sensitivity and specificity of 80% and 85% respectively, affirming SWE could help identify which patients should be treated surgically (106);
- in 2018, Chang et al. published a meta-analysis with 20 papers and 3397 thyroid nodules, assessed by quantitative SWE. They showed a sensitivity and specificity of 68 and 85% concluding that SWE is very accurate in distinguishing malignant and benign nodules (107);
- in 2020, Filho et al. published a meta-analysis with 17 papers and 3,806 thyroid nodules (2,428 benign and 1,378 malignant) assessed by 2D–SWE elastosonography by various manufacturers. They showed a sensitivity and specificity of 77 and 76% respectively for T–SWE (Toshiba shear-wave elastography); a sensitivity and specificity of 72 and 81% respectively for VTIQ (Virtual Touch tissue imaging and Quantification); and a sensitivity and specificity of 63 and 81% respectively for S-SWE (SuperSonic shear-wave elastography). In conclusion they affirm that 2D–SWE could rule out the malignant thyroid nature (108).

Furthermore, some meta-analyses which compare these two different elastosonographic techniques were published:

- in 2017, Hu et al. published a meta-analysis with 22 original articles and 2,661 thyroid nodules (2,063 benign and 598 malignant) assessed by SE (Strain Elastography) and SWE. They showed a sensitivity and specificity of 84 and 90% respectively for SE and a sensitivity and specificity of 79 and 87% respectively for SWE. In conclusion, they state that SE has a better sensitivity than SWE (0.84 vs. 0.79) with p-value <0.05 and above all a statistically better specificity than SWE (0.90 vs. 0.87 with p <0.05) (109);
- in 2016, Tian et al. published a meta-analysis with 54 papers with 10,001 thyroid nodules (7,380 benign and 2,621 malignant) assessed by SE (Strain Elastography) and SWE. They showed a sensitivity and specificity of 82.9 and 82.8% respectively for SE, and sensitivity and specificity of 78.4 and 82.4% for SWE. In conclusion they affirm that the SE sensitivity is better than SWE one (0.829 vs. 0.784) but with similar specificity (103).

The USE role is not limited to the thyroid cancer diagnosis but it is also useful in the detection of cervical lymph node metastases and to guide interventistic procedures (110). In fact, the EFSUMB guidelines state that USE can identify the most suspicious lymph nodes and the most suspicious internal areas worthy of cyto-histological investigation (92).

Our meta-analysis is the first meta-analysis since 2016 that individually takes into consideration the diagnostic performance of different USE types in the characterization of the thyroid nodule however using studies with at least 50 thyroid nodules because the smaller ones may have low precision (wide confidence interval of the estimates), may be of low quality and may increase heterogeneity.

In particular we examined qualitative USE, semiquantitative USE and quantitative USE and demonstrate that all of them are useful in the thyroid nodule characterization with high accuracy values and especially the same specificity. However, the semiquantitative and qualitative elastosonography showed the best diagnostic performance compared to SWE with the following sensitivity and specificity values 84 and 81% for qualitative USE, 83 and 80% for semiquantitative USE and 78 and 81% for quantitative USE.

Our results about strain-based USE techniques are similar with no significant statistical difference (p-value >0.05).

By contrast, the AUCs evaluation slightly favors the SRE over others (semiquantitative USE AUC: 0.93; qualitative USE: 0.89; quantitative USE: 0.87) with statistically significant values between semiquantitative USE and quantitative USE (p-value <0.05).

Our metanalysis results are quite in line with this recent meta-analyses and guidelines that indicate SRE the most accurate USE method in the malignancy risk stratification of the thyroid nodules (10).

Although in 2017 the Hu et al. meta-analysis showed the better qualitative USE sensitivity and specificity than SWE ones (0.84 vs. 0.79 with p >0.05 and 0.90 vs. 0.87 with p <0.05, respectively), the semiquantitative USE was poorly represented in their paper and not distinguished from qualitative USE in the statistical analysis (109).

In 2016 the meta-analysis of Tian concluded asserting that the SE (Strain Elastography) diagnostic performance (both qualitative and semiquantitative USE) was better than SWE with a p <0.05 and among the SE techniques the SRE (strain ratio elastography) accuracy was better than SE with elasticity score with sensitivity and specificity values of 86.5% vs 81.8% and 86.6% vs 81.7% (103).

These differences could be explained because the main qualitative USE limitation is the operator-dependence related to the subjective diagnostic evaluation based on different eye-type scales without agreement about the score to be used (55). In literature, several qualitative USE color pattern involving five, four, or two color score are used, but showing different diagnostic performances without having a better one (55).

SRE improves the subjective assessment of the nodule stiffness, in some cases it is not feasible due to the presence of micro-macrocalcifications, pathological changes in the surrounding parenchyma such as in autoimmune thyroiditis or when the nodule is so large as to replace the entire gland without healthy parenchyma to compare. Furthermore, there is no agreement on the SRE cut-off to choose, and therefore without having a real standardization of this method.

SWE is the quantitative USE technique based on the Shear-wave speeds measurement and so less affected by a subjective interpretation. But to date, the current and recent papers showed a worse SWE diagnostic accuracy than SE one. SWE can evaluate thyroid nodule also in presence of autoimmune thyroiditis (111) and so when SE is unfeasible for the pathological changes of peri nodular surrounding thyroid parenchyma.

I^2^ quantify the effect of heterogeneity, describing the percentage of total variation across studies that is due to heterogeneity rather than chance. In our results it is very high for all parameters (>80%) except for the sensitivity of the SWE which is instead about 55.1%. This could lead to think that it is a technique less influenced by interobservational variability but nevertheless its I^2^ is too high (>50%) and not low enough to affirm a good homogeneity between the different studies. One explanation could be that the qualitative and semi-quantitative USE techniques have been used for a long time and therefore the resulting studies are older and more heterogeneous. Other reasons should explain it such as a possible more homogeneous population or settings. The interobservational variability of the different USE techniques is beyond our purposes but to date it has been evaluated by few studies. Therefore, further studies are needed, especially prospective and with a large population.

In our study there are some limitations: at first, calcified and/or cystic nodules are not included by some studies for possible artifacts generation; secondly, the heterogeneity of the articles included may represent a source of bias as no consensus about the optimal elastosonographic methodology as the preferential use of carotid or freehand pulsation in the strain elastography; the non-univocal qualitative USE score to use (score 1–2; score 1–4 or score 1–5) and different Strain Ratio cut-off values; thirdly, the possible selection bias. In fact our thyroid nodules population presents a high pooled malignancy rate (33%) deriving from the studies published by various research institutes considered as a reference center for thyroid pathology and so with many patients with already suspected thyroid nodule. All this might have contributed to have misleading results.

In addition, we have to mention that we did not evaluate the inter-observational variability between the different USE techniques and secondly specific papers on indeterminate nodules at FNAC have not been.

Noteworthy, although FNAC is the gold standard for the thyroid nodule classification, it can show cellular atypia of undetermined significance (TIR3 category) in the 5–20% of cases (112). Therefore a fairly large number of patients undergo thyroidectomy for diagnostic rather than therapeutic purposes, with increased costs and possible complications. Therefore, in recent years, efforts have been made to better evaluate the cytologically indeterminate nodule and reduce the number of these thyroidectomies as only up to 30% of these patients harbor indeed thyroid cancer.

In this regard, MPUS tries to better characterize indeterminate thyroid nodules and with encouraging results (96, 106, 107, 113).

## Conclusions

In conclusion, this comprehensive meta-analysis shows that all USE methods (quantitative, semi-quantitative and quantitative USE) have a good sensitivity and specificity in differentiating malignancy from benignancy, with a slight better performance by means of qualitative USE.

## Data Availability Statement

The raw data supporting the conclusions of this article will be made available by the authors, without undue reservation.

## Author Contributions

Conceptualization, VC, VDA, and CC Methodology, VC, DF, VS, ADS, CD, and GG Investigation, ADS, VS, DF, PP, EP, GP, and OG Resources, SS, PT, CD, and MT Data curation, DF, VC, CD, GG, and PT Writing—original draft preparation, DF, CD, VC, AL, RC, OG, and PT Writing—review and editing, VC, AMI, CD, GG, and EG Supervision, VC, CC, VDA, PT, and CD All authors listed have made a substantial, direct, and intellectual contribution to the work and approved it for publication.

## Conflict of Interest

Author VC reports a lecturer fee from Bracco, Samsung and Toshiba.

The remaining authors declare that the research was conducted in the absence of any commercial or financial relationships that could be construed as a potential conflict of interest.​

## Publisher’s Note

All claims expressed in this article are solely those of the authors and do not necessarily represent those of their affiliated organizations, or those of the publisher, the editors and the reviewers. Any product that may be evaluated in this article, or claim that may be made by its manufacturer, is not guaranteed or endorsed by the publisher.
